# Frequency and Clinical Impact of Family History of Coronary Artery Disease in Patients with Vasospastic Angina

**DOI:** 10.3390/jcdd10060249

**Published:** 2023-06-08

**Authors:** Hiroki Teragawa, Yuko Uchimura, Chikage Oshita, Yu Hashimoto, Shuichi Nomura

**Affiliations:** Department of Cardiovascular Medicine, JR Hiroshima Hospital, 3-1-36, Futabanosato, Higashi-ku, Hiroshima 732-0057, Japan; yuuko-uchimura@jrhh.or.jp (Y.U.); chikage-ooshita@jrhh.or.jp (C.O.); yu-hashimoto@jrhh.or.jp (Y.H.); shuuichi-nomura@jrhh.or.jp (S.N.)

**Keywords:** coronary spasms, coronary spastic angina, family history of coronary artery disease, peripheral vascular function, vasospastic angina

## Abstract

Background: Family history (FH) of coronary artery disease (CAD) [FH-CAD] is a well-known risk factor for atherosclerotic CAD. However, FH-CAD frequency in patients with vasospastic angina (VSA) remains unknown, and the clinical characteristics and prognosis of VSA patients with FH-CAD are unclear. Therefore, this study compared FH-CAD frequency between patients with atherosclerotic CAD and those with VSA and examined the clinical characteristics and prognosis of VSA patients with FH-CAD. Methods: Coronary angiography and spasm provocation tests (SPT) were used to investigate chest pain of coronary artery origin in patients classified into atherosclerotic CAD (362 cases), VSA (221 cases; positive for SPT) and non-VSA (73 cases; negative for SPT) groups, with FH-CAD being defined. In the VSA group, flow-mediated vasodilation (FMD) and nitroglycerin-independent vasodilation (NID) via brachial artery echocardiography and clinical symptoms in the groups with and without FH-CAD were checked, with Kaplan–Meier curves revealing major adverse cardiovascular events (cardiac death and rehospitalisation for cardiovascular disease) between the two groups. Results: The atherosclerotic CAD group had a significantly lower FH-CAD frequency (12%, *p* = 0.029) than the VSA (19%) and non-VSA groups (19%). FH-CAD was more common in females in the VSA and non-VSA groups than in the atherosclerotic CAD group (*p* < 0.001). Nonpharmacological treatment for CAD in FH-CAD was more common in the atherosclerotic CAD group (*p* = 0.017). In the VSA group, FH-CAD tended to be more common in females (*p* = 0.052). Although no differences in FMD of the brachial artery were observed between the groups, the FH-CAD (+) group had significantly higher NID than the FH-CAD (−) group (*p* = 0.023). Kaplan–Meier’s analysis revealed a similar prognosis between the two groups, and other clinical characteristics did not differ. Conclusion: Patients with VSA have a higher FH-CAD frequency than those with atherosclerotic CAD, especially in females. Although FH-CAD may affect vascular function in patients with VSA, its effect on the severity and prognosis of VSA appears to be minimal. FH-CAD and its confirmation may assist in CAD diagnosis, especially in female patients.

## 1. Introduction

Vasospastic angina (VSA) is a disease in which epicardial coronary arteries constrict, resulting in myocardial ischemia that induces resting angina pectoris or exertional angina, acute coronary syndrome, heart failure and sudden death [1,2,3,4,5,6]. Recently, more emphasis has been placed on the aetiology, diagnosis and treatment of VSA as a cause of ischemia and myocardial infarction with nonobstructive coronary artery disease (CAD). Several risk factors, including serum biomarkers, for coronary spasms have been reported [7,8,9], among which smoking is considered an important risk factor [3,10,11].

Family history (FH) of CAD [FH-CAD] is a risk factor for atherosclerotic CAD [12,13,14,15,16,17], and some reports have demonstrated that FH-CAD may also be a risk factor for VSA, especially in female patients [18,19,20]. However, studies comparing FH-CAD frequency between patients with VSA and those with atherosclerotic CAD while exploring FH-CAD effects on the clinical characteristics and prognosis of patients with VSA remain scarce. Therefore, this study investigates whether the frequency of FH-CAD in patients with VSA differs from that in patients with other CAD types, especially atherosclerotic CAD, among patients who underwent coronary angiography in our hospital, and how it affects the clinical characteristics and prognosis in patients with VSA.

## 2. Materials and Methods

### 2.1. Study Population

**Figure 1:**
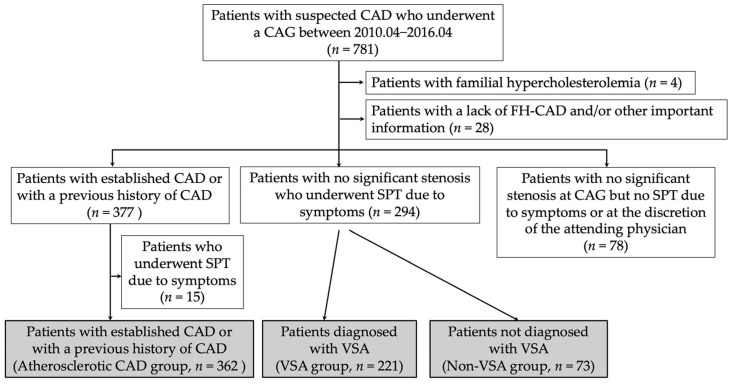
Study flowchart. CAD, coronary artery disease; CAG, coronary angioography; FH-CAD, family history of coronary artery disease; SPT, spasm provocation test; VSA, vasospastic angina.

This was an observational and retrospective study involving 781 patients who underwent coronary angiography (CAG) for the evaluation of possible CAD-related chest pain. Initially, cases of aortic stenosis and other valvular diseases, as well as cases of dilated, hypertrophic, restrictive and takotsubo cardiomyopathies performed to exclude CAD, were excluded from the study. For patients undergoing repeat CAG, the initially obtained data were used. Patients with familial hypercholesterolemia (*n* = 4) were excluded from the study. The electrocardiogram revealed no cases of Brugada syndrome or QT prolongation syndrome. Cases with missing FH or blood test data were also excluded (*n* = 28). Among the remaining 749 patients, 78 patients without significant atherosclerotic stenosis on CAG did not undergo a spasm provocation test (SPT) due to symptoms or at the discretion of the attending physician, and they were excluded. There were 377 patients with established CAD (significant coronary stenosis with any findings suggestive of myocardial ischemia) or with a previous history of CAD and 294 patients with no significant coronary stenosis who underwent SPT due to their symptoms. Fifteen patients with significant coronary stenosis who had been diagnosed with coronary spasms after SPT based on their symptoms were also excluded from the study. The final analysis was performed on 656 patients (408 males and 248 females; mean age, 68 years; Figure 1). Based on the results of CAG and/or SPT, 656 patients were divided into three groups: atherosclerotic CAD group (*n* = 362), VSA group (*n* = 221) and non-VSA group (*n* = 73) (Figure 1).

The study protocol was approved by the ethics committee of our institution. Written informed consent was obtained from all patients.

### 2.2. Coronary Angiography (CAG) and Spasm Provocation Test (SPT)

Patients with documented coronary stenosis detected via cardiac computed tomography, positive ischemic findings discovered via exercise stress electrocardiograms (ECG) or pharmacological stress myocardial perfusion imaging or typical ischemic chest symptoms during exercise continued to take all prescribed medications. Using radial artery or brachial artery approaches, a diagnostic CAG was conducted using 4-French gage (4-Fr) or 5-French Judkins diagnostic catheters. In addition, 300-µg nitroglycerin (NTG) was administered intravenously or intracoronarily immediately following the sheath’s placement. An autoinjector (ZoneMaster, Sheen Man, Osaka, Japan) capable of injecting 5 mL of contrast media at 2.5 mL/s was used to create an angiogram from several projections. After an intravenous infusion of adenosine triphosphate, we measured the fractional flow reserve (FFR) to determine the functionality of organic stenosis in cases where a moderate atherosclerotic lesion was identified on angiograms (Prime-Wire Prestige Plus Guide Wire or Verrata Pressure Guide Wire, Phillips Volcano Therapeutics Inc., Rancho Cordova, CA, USA) [21].

In patients with suspected VSA or with chest symptoms at rest alone, during exercise, or both at rest and during exercise, except for sublingual NTG, which was only withheld starting 1 h before catheterisation, all antianginal medications were stopped at least 48 h prior to catheterisation. Using the percutaneous brachial route, we conducted an SPT following the usual diagnostic CAG. Through inserting it through the medial cubital vein or internal jugular vein, a 5-Fr transient pacing catheter (Bipolar Balloon Catheter, Bebrawn, Melsungen, Germany) was set to 50 beats per minute. Using a multichannel recorder, continuous monitoring and recording of arterial pressure, heart rate and ECG measurements were performed (Polygraph 1600, Nihon Electric Corporation, Tokyo, Japan).

The conduct of routine SPT has been previously described [22,23,24]. Briefly, after the initial CAG, acetylcholine (ACh) doses of 20 or 50 µg were injected into the right coronary artery (RCA) for 20 s, with 3 min breaks in between doses. If the 50 µg dose failed to induce coronary spasms, a maximum of 80 µg ACh was injected into the RCA. The highest ACh infusion or induction of coronary spasms was followed by instantaneous CAG. After inducing a spasm in the RCA, we injected 50 or 100 µg ACh into the left coronary artery (LCA) for 20 s, with 3 min breaks in between doses. A maximum of 200 µg ACh was injected into the LCA, with or without 20, 40 or 60 µg of methylergometrine, if 200 µg ACh induced no coronary spasms. The highest ACh infusion or induction of coronary spasms was followed by instantaneous CAG. We completed the last CAG of the LCA after intracoronary administration of 300 µg NTG. If the patient’s haemodynamics remained unstable, small doses of catecholamines were given intravenously or via the coronary artery. The RCA and LCA received 20 µg and 50 µg, respectively, of low dosages of ACh (L-ACh) in this investigation.

### 2.3. Definition of CAG and Coronary Spasm-Related Factors

Strategies for measuring coronary artery diameters have been described in [23,24]. For a quantitative investigation, we selected spastic and atherosclerotic segments. A single researcher who was blinded to the clinical information evaluated the luminal diameters in each case using an end-diastolic frame in a computer-assisted coronary angiographic analysis system (CAAS II/QUANTCOR; Siemens, Berlin, Germany). Measurements were made three times, and analysis was performed using the average value. Changes in coronary artery diameter induced via ACh and NTG infusions were expressed as percentage changes from baseline angiographic data.

Lesions with stenosis exceeding 20% were classified as atherosclerotic lesions. Organic stenosis was defined as a percent stenosis value exceeding 50% and/or an FFR value of 0.8. We looked for myocardial bridging (MB) [25,26], a well-known associator of VSA. An MB was defined as a systolic narrowing of the coronary artery diameter by more than 20% compared to that in diastole [27]. Variant angina (VA) was defined as angina with an ECG spontaneous ST elevation. VSA was identified during the SPT as 90% constriction of the coronary arteries on angiograms, as well as the presence of characteristic chest pain and/or an ST-segment deviation on the ECG [28]. Multivessel spasm (MVS) was characterised as coronary spasms involving two major coronary arteries, whereas single-vessel spasms were classified as involving just one major coronary artery. Because we could not ascertain whether the SPT obtained a negative result in another coronary artery after the necessary use of NTG in one coronary artery with coronary spasms during the SPT, such patients were excluded from the analysis of the frequency of MVS. According to the American Heart Association, a focal spasm is characterised by a transitory vessel narrowing of >90% within the boundaries of a single isolated coronary segment. A 90% diffuse vasoconstriction in two adjacent coronary segments of the coronary arteries was used to characterise a diffuse spasm [29]. Focal spasms are considered one of the poor prognostic factors in VSA [29,30], and this study investigated the frequency of focal spasms. A focal spasm was considered to have occurred in either coronary artery in the instance of MVS. The following characteristics were also examined: the frequencies of L-ACh-induced spasms, total occlusion (TOC) of a coronary artery caused by spasms and ST elevation on ECG.

### 2.4. Definitions of FH-CAD

FH-CAD was determined through interviewing patients and their family members or through using information from their electronic medical records. The first question asked whether any family members (first-degree relatives) had suffered from angina pectoris or myocardial infarction or had died suddenly of heart problems, thus confirming the presence of FH-CAD in first-degree relatives [17]. Second, even if the information was unavailable due to parental divorce or if parents did not have such FH-CAD, second-degree relatives, especially siblings, were asked if there were two or more patients from angina, myocardial infarction or sudden cardiac death, and such second-degree relatives were included in the FH-CAD in this study. A history of non-pharmacologic treatment for CAD, such as percutaneous coronary intervention or coronary artery bypass graft, and probable sudden death of presumed cardiac origin were also investigated in first- and second-degree relatives. Some of the sudden deaths were unclear to be of cardiac origin based on the interview, but if information was obtained during the interview that the sudden death was due to cerebrovascular disease, that information was adopted and excluded from the FH-CAD in this study. The FH of premature onset of CAD (males aged <55 years and females aged <65 years) was not adopted in this study because patients and other family members could not accurately determine the age of onset of the disease of relatives or because we did not check the age of onset in some of the cases. Nevertheless, the onset of CAD and sudden cardiac death in clearly elderly patients (aged ≥ 75 years) were excluded from our definition of FH-CAD.

### 2.5. Parameters

The patients’ current smoking behaviour, especially as active smokers, was documented. Alcohol consumption was defined as drinking at least once per week [31]. A systolic blood pressure of at least 140 mmHg, a diastolic blood pressure of at least 90 mmHg or the uses of antihypertensive medication were all considered indicators of hypertension. Before CAG, blood chemistry parameters were examined in a fasted state in the morning. The existence of chronic kidney disease (CKD) was determined using accepted criteria, and the estimated glomerular filtration rate (eGFR, mL/min/1.73 m^2^) was computed using the accepted formula [32]. A low-density lipoprotein cholesterol level of less than 120 mg/dL or the use of drugs for dyslipidaemia was considered dyslipidaemia. A fasting blood sugar level of more than 126 mg/dL, an Hb A1c level morethan 6.5% or the use of antidiabetic drugs were considered indicators of diabetes mellitus (DM). In order to calculate the left ventricular ejection fraction (LVEF), cardiac ultrasonography was used. As previously mentioned [33,34], the functions of endothelium-dependent flow-mediated dilatation (FMD) and endothelium-independent NTG-induced dilation (NID) were evaluated.

The frequency of VA or other significant symptoms (such as cold sweating and syncope) as well as anginal attacks that happened at rest or after physical exercise were also examined as associated parameters of VSA. Although only one angina attack occurred in some cases, the total number of angina attacks was determined through estimating the frequency of angina episodes each month between the onset of chest discomfort and hospital admission. The time of onset of the anginal attack was estimated and the time to hospitalisation was used as the estimated diseased duration.

After discharge, patients received follow-up care at our facility, with at least one follow-up visit for each study participant. Follow-up assessments included medication details recorded from patients’ medication diaries, as well as the number of angina events and use of coronary vasodilators in the previous three months (per month). The final data collection was planned to end in March 2023. The number of coronary vasodilators was assessed at the last follow-up. Cardiac events were tracked, including readmission for angina or other cardiovascular problems, and major adverse cardiovascular events (MACEs) were defined as death from cardiac causes or readmission for cardiovascular reasons [24,33,34].

### 2.6. Statistical Analyses

Mean and standard deviation were used to convey regularly distributed data, while median was used to express normally distributed but non-continuous data (interquartile range). Using the Wilcoxon signed-rank test, Student’s unpaired t-test or Chi-square test, the groups’ initial characteristics were compared. The Kaplan–Meier survival curve and log-rank test were used to analyse MACEs. All statistical analyses were performed utilising JMP Ver. 16 (SAS Institute Inc., Cary, NC, USA). The cutoff for statistical significance was a *p* value of 0.05.

## 3. Results

### 3.1. Patients’ Characteristics and Frequency of FH-CAD of the Three Groups

The atherosclerotic CAD, VSA and non-VSA groups included 362, 221 and 73 patients, respectively (Figure 1). Table 1 presents the patients’ characteristics. While the atherosclerotic CAD group had older patients than the VSA and non-VSA groups, it had significantly higher frequencies of men, dyslipidaemia, DM and CKD and significantly lower LVEF on echocardiography. Active smokers were less frequent in the non-VSA group (*p* = 0.012). Notably, body mass index and hypertension did not differ significantly among the three groups (Table 1).

**Figure 2:**
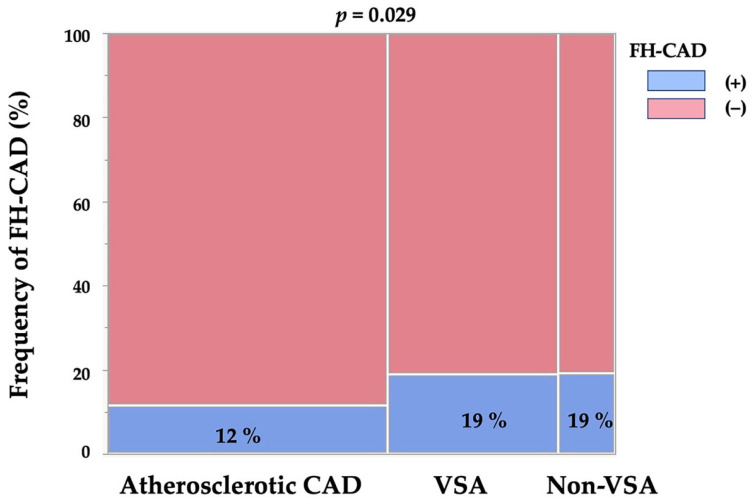
FH-CAD frequencies in the three groups. CAD, coronary artery disease; FH-CAD, family history of coronary artery disease; VSA, vasospastic angina.

Table 2 lists the obtained FH-CAD frequencies, which were 12%, 19% and 19% in the atherosclerotic CAD, VSA and non-VSA groups, respectively, with a significantly lower incidence of FH-CAD in the atherosclerotic CAD group (*p* = 0.029, Figure 2). The VSA and non-VSA groups had comparable FH-CAD frequencies.

When only patients with FH-CAD were included in the analysis, the three groups had different frequencies of male and female patients (*p* < 0.001), with the atherosclerotic CAD group having a higher frequency of male patients. The frequency of first-degree relatives only, second-degree relatives only and sudden cardiac death did not differ between the three groups. However, the atherosclerotic CAD group had a greater frequency of nonpharmacologic treatment for CAD than the other two groups (*p* = 0.017, Table 2).

### 3.2. Clinical Characteristics and Prognosis in VSA Patients Based on the Presence of FH-CAD

As noted above, FH-CAD was present in 42 patients with VSA (19%, FH-CAD (+) VSA group) and absent in 179 patients (81%, FH-CAD (−) VSA group). Table 3 presents the clinical characteristics in the two groups. Age did not differ significantly between the two groups, although a trend existed toward more female patients (*p* = 0.052). No significant differences were observed in conventional coronary risk factors, CKD frequency, or LVEF between the two groups.

As determined via brachial ultrasonography, the brachial artery diameter at baseline and FMD did not significantly differ between the two groups; however, NID was significantly greater in the FH-CAD (+) group than in the FH-CAD (−) group (*p* = 0.023). While both groups showed no difference in other symptoms related to coronary spasms, including occurrence time, estimated duration, number of monthly anginal pain and VA frequency, they also did not differ in the frequency of atherosclerotic lesions or MB in CAG. Notably, no difference was observed in the frequency of coronary spasms induced via L-ACh, TOC, focal spasms, MVS, ST elevation during SPT and unavoidable use of NTG during SPT (Table 4).

### 3.3. Prognosis in VSA Patients Based on FH-CAD

**Figure 3:**
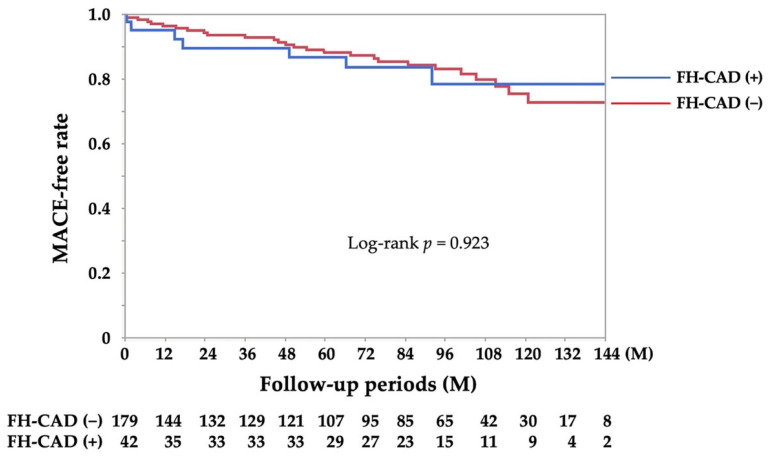
Kaplan–Meier curve for MACE-free survival during the follow-up period for the FH-CAD (+) and FH-CAD (−) groups. FH-CAD, family history of coronary artery disease; M, month; MACE, major adverse cardiac event.

The median (interquartile range) follow-up period was 88 (45, 112) months. The average number of monthly anginal attacks and number of coronary vasodilators at the final follow-up were not different between the two groups (*n* = 135 and 35 in the FH-CAD (−) and FH-CAD (+) groups, respectively; Table 4). The thirty-four confirmed MACE cases included one for cardiac arrest, six for heart failure, four for percutaneous coronary intervention, eighteen for rehospitalisation due to unstable angina, one for acute myocardial infarction, one for mitral valve plasty, one for the need of an implantation of a permanent pacemaker, one for endovascular therapy for lower extremity arterial disease and one for cerebrovascular disease. No difference was observed in the frequency of MACEs between the two groups (log-rank, *p* = 0.923; Figure 3).

## 4. Discussion

This study examined FH-CAD frequency among patients diagnosed with VSA and determined whether any differences existed in terms of frequency and characteristics when compared to patients with atherosclerotic CAD. Patients with VSA had a higher FH-CAD frequency than those with atherosclerotic CAD, with this frequency being more prevalent in female patients. The FH-CAD content also varied between the two groups. While female patients with VSA tended to exhibit a higher FH-CAD frequency than their male counterparts, peripheral ultrasonography revealed that VSA patients with FH-CAD responded better to NTG. Furthermore, the study demonstrated that FH-CAD is not an indicator of coronary spasm severity or prognosis. In patients with possible coronary spasms, especially female patients, FH-CAD and its description during the interview may be clues for VSA diagnosis.

FH-CAD, especially young-onset CAD, is undeniably a risk factor for atherosclerotic CAD [12,13,14,15,16,17]. According to research, 12.2% of patients over the age of 20 had a parent or sibling who had suffered a heart attack or angina pectoris before turning 50 [35]. A significant issue with FH-CAD is its lack of a uniform definition, and its frequency depends on its definition [17]. Its frequency may also differ based on how the target patient population is defined and whether it is limited to younger age groups [36]. Regarding VSA, several studies have demonstrated the importance of FH-CAD in patients with VSA [18,19,20,29,37], with many finding FH-CAD frequency in the 10% range [18,19,29,37], although this frequency seems to exceed 52% when the definition of FH-CAD that includes stroke is used [20]. Until now, only a few studies have compared FH-CAD frequency in patients with atherosclerotic CAD and VSA. The definition employed in the present study included not only first-degree relatives but also second-degree relatives with CAD or sudden cardiac death, constituting a rather broad definition. Certainly, this expansive definition could have yielded a higher FH-CAD frequency than those reported previously [18,19,29,37]. However, the strength of our study lies in its use of a uniform definition, which revealed a higher FH-CAD frequency in patients with VSA than in those with atherosclerotic CAD. In addition, we reported the sex differences as well as differences in FH-CAD content when comparing the two groups. While patients with atherosclerotic CAD had more nonpharmacological treatments in their FH, the VSA and non-VSA groups had fewer nonpharmacological treatments in their FH-CAD. While interviewing about FH-CAD, we confirmed not only the presence or absence of FH-CAD but also the sex difference in relatives with CAD and the treatment received by relatives with CAD. This information for FH-CAD may help differentiate the presence of atherosclerotic CAD or coronary artery dysfunction, such as VSA. Future studies are needed to confirm the novelty of this study in a multi-centre registry or in other studies.

Despite the abundance of studies on the heritability of VSA, including polymorphisms in eNOS [38,39] and aldehyde dehydrogenase 2 [40,41], sex differences in abnormalities of these genes remain unexplored. VSA patients with and without FH-CAD showed no significant sex differences (*p* = 0.052), although a trend toward a higher FH-CAD frequency in females existed, corroborating previous clinical findings [18,19,20]. Although VSA pathogenesis may be diverse and not singly deterministic, the possibility of a hereditary component, especially in women, is an important finding in VSA diagnosis. Notably, FH-CAD effects on the clinical manifestations of VSA are unknown. In the present study, although not all patients were evaluated, the subject group examined for brachial artery peripheral vascular function showed no difference in FMD, but a significant increase in NID was observed. Whether this indicates relative vascular endothelial dysfunction or vascular smooth muscle dysfunction at baseline is unclear, but some vascular dysfunction may be present in VSA patients with FH-CAD. Further studies are needed to clarify the relationship between FH-CAD and vascular function in patients with VSA in a multi-centre registry and/or the relationship between genetic abnormalities in eNOS, FH-CAD and vascular dysfunction in specialised centres. While studies have not explored the FH-CAD effect on the prognosis of patients with VSA [19,29,37,42], our study examined reported prognostic factors for coronary spasms, including VA, focal spasms and MVS [24,29,30,42], but did not find any association between FH-CAD and these prognostic factors. Based on the Kaplan–Meier curve for the MACE-free event rate, we concluded that an insignificant association existed between FH-CAD and prognosis in patients with VSA.

The present study also showed that the FH-CAD frequency in patients without significant stenosis and negative SPT (non-VSA group) was similar to that in the VSA group. The frequency of FH in sudden cardiac death may be lower in the non-VSA group (7%), although this difference was not significant compared with the VSA group (26%). Although the number of cases in the non-VSA group was quite small to allow for sufficient analysis, the occurrence of sudden cardiac death might help in differentiating patients with VSA from those without VSA. On the contrary, some studies have reported a higher FH-CAD frequency in patients with VSA than in those without VSA [29,37,43]. This discrepancy may be due to differences in the definition of FH-CAD or in the characteristics of the studied patients (a paper [43] examined coronary spasms and FH-CAD in patients with MINOCA). In addition, the non-VSA group did not undergo coronary microvascular function tests, which are commonly administered these days [44], during this study. Therefore, important pathological types such as microvascular spasms and/or coronary microvascular dysfunction (CMD) could not be differentiated [28,45]. Given the existence of a relationship between FH-CAD and CMD [46], future studies are needed clarify the FH-CAD effect on clinical symptoms and prognosis of microvascular spasms and/or CMD after differentiating the pathologic type.

This study has several limitations. First, it was conducted using a small sample size and was retrospectively performed at only one institution, raising questions about the generalisability of the results. Second, FH-CAD lacks a standard definition [17]. The definition of FH-CAD often uses cases occurring in the first degree of consanguinity, but we included cases in the second degree of consanguinity due to the potential for heritability. The variability in the FH-CAD definition, which affects its frequency [17], highlights the need for standardised definitions in future research. The reliability of FH-CAD appears to be decreasing [47] due to difficulties in rigorously examining FH in clinical research. In addition, the age of onset is often ambiguously defined as being in the 50s or 60s and because of our lack of confirmation of the age of onset, evaluating premature FH-CAD accurately in this study is difficult. Third, although coronary spasms can occur even with organic stenosis in daily clinical practice [48], such cases were excluded from this study (*n* = 15) because the frequency of FH-CAD in patients between atherosclerotic CAD and VSA was separated. However, we only excluded patients who had atherosclerotic CAD and a positive SPT result based on symptoms, such as chest pain at rest. Previous studies have also reported coronary spasms after implantation of drug-eluting stents [28,49]. Thus, this group of patients with atherosclerotic CAD will test positive for coronary spasms if they undergo SPT. In addition, 78 patients without significant coronary stenosis were excluded based on their symptoms or according to the judgment of their attending physicians. However, if we had conducted an SPT in this group, they would have been included in the VSA and non-VSA groups, and the results might have been different. Fourth, the Achilles tendon was touched but not measured, and patients with familial hypercholesterolemia and Brugada syndrome could not be ruled out based on a single ECG because the ECG may change from day to day. Fifth, the Seattle Angina Questionnaire and other methods for evaluating the quality of chest pain are available, but we could not evaluate them in this study. Finally, insufficient prognostic information may have been collected since we failed to follow the patients closely enough.

## 5. Conclusions

We investigated FH-CAD frequency in patients with VSA and determined whether any differences existed in terms of frequency and characteristics when compared to patients with atherosclerotic CAD. Patients with VSA exhibited a higher FH-CAD frequency than patients with atherosclerotic CAD, with this frequency being more prevalent in female patients. The FH-CAD content also differed between the two groups. While female patients with VSA tended to exhibit a higher FH-CAD frequency than their male counterparts, peripheral ultrasonography revealed that VSA patients with FH-CAD responded better to NTG. Notably, this study revealed that FH-CAD is not an indicator of coronary spasm severity or prognosis. FH-CAD and its description during the interview may aid in VSA diagnosis in patients with possible coronary spasms, especially female patients. A multi-centre registry should confirm the effect of FH-CAD on its frequency and clinical manifestations in patients with CMD and VSA.

## Figures and Tables

**Table 1:** Patients’ characteristics among the three groups.

		Atherosclerotic CAD	VSA	Non-VSA	*p* Value
No.		362	221	73	
Age (years)		69 ± 11	67± 11	66 ± 13	0.004
Male/Female		270/92	114/107	24/49	<0.001
Body mass index		24.2 ± 4.7	24.2 ± 4.2	24.4 ± 3.8	0.933
Coronary risk factors (%)					
	Active smoker	86 (24)	47 (21)	6 (8)	0.012
	Hypertension	264 (73)	158 (71)	50 (68)	0.731
	Dyslipidaemia	294 (81)	138 (62)	42 (58)	<0.001
	Diabetes mellitus	159 (45)	49 (22)	15 (21)	<0.001
CKD (%)		188 (53)	66 (30)	25 (35)	<0.001
LVEF on UCG (%)		62 ± 12	66 ± 9	68 ± 6	<0.001

Numbers were expressed as the numbers (percentage) and values were expressed as the mean with standard deviation. CAD, coronary artery disease; CKD, chronic kidney disease; LVEF, left ventricular ejection fraction; No., number; UCG, ultrasonic echocardiography; VSA, vasospastic angina.

**Table 2:** FH-CAD in the three groups.

		Atherosclerotic CAD	VSA	Non-VSA	*p* Value
FH-CAD (No., %)		42 (362,12)	42 (221, 19)	14 (73, 19)	0.029
	Male patients (No., %)	33 (270, 12)	16 (114, 14)	4 (24, 17)	0.764
	Female patients (No., %)	9 (92, 10)	26 (107, 24)	10 (49, 20)	0.027
Patients with FH-CAD					
	No.	42	42	14	
	Male/Female	33/9	16/26	4/10	<0.001
	First-degree relative (%)	31 (74)	31 (74)	10 (71)	0.983
	Only second-degree relative (%)	11 (26)	11 (26)	4 (29)	0.983
	Non-pharmacological therapy for CAD (%)	24 (52)	12 (29)	4 (29)	0.017
	Probable sudden cardiac death (%)	7 (17)	11 (26)	1 (7)	0.248

Numbers were expressed as the numbers (percentage). CAD, coronary artery disease; FH-CAD, family history of coronary artery disease; No., number; VSA, vasospastic angina.

**Table 3:** Clinical characteristics in VSA patients with/without FH-CAD.

		FH-CAD (−) VSA Group	FH-CAD (+) VSA Group	*p* Value
No. (%)		179 (81)	42 (19)	
Age (years)		68 ± 11	65 ± 12	0.178
Male/Female		98/81	16/26	0.052
Body mass index		22.4 ± 4.1	24.2 ± 4.5	0.978
Coronary risk factors (%)				
	Active smoker	39 (22)	8 (19)	0.696
	Hypertension	128 (72)	30 (31)	0.992
	Dyslipidaemia	112 (63)	26 (62)	0.936
	Diabetes mellitus	40 (22)	9 (21)	0.898
Alcoholic drinker (%)		73 (41)	11 (26)	0.080
CKD (%)		51 (28)	15 (36)	0.357
LVEF on UCG (%)		66 ± 10	68 ± 6	0.364

Numbers were expressed as the numbers (percentage) and values were expressed as the mean with standard deviation. CKD, chronic kidney disease; FH-CAD, family history of coronary artery disease; LVEF, Left ventricular ejection fraction; No., number; UCG, echocardiography; VSA, vasospastic angina.

**Table 4:** The results of brachial ultrasonography and VSA-related parameters in VSA patients with/without FH-CAD.

		FH-CAD (−) VSA Group	FH-CAD (+) VSA Group	*p* Value
Brachial ultrasonography				
	No.	152	40	
	Brachial artery diameter at baseline (mm)	3.9 ± 0.6	3.7 ± 0.7	0.073
	FMD (%)	3.9 ± 3.1	3.4 ± 3.0	0.384
	NID (%)	14.0 ± 6.0	16.7 ± 8.4	0.023
VSA-related symptoms				
	At rest/on exercise/both	146/18/15	34/6/2	0.554
	Estimated diseased period (M)	6 (2, 20)	5 (2, 36)	0.518
	No. of anginal attack/M	4 (2, 10)	5 (2, 10)	0.321
	VA (%)	6 (4)	0 (0)	0.229
CAG				
	Atherosclerosis (%)	109 (61)	23 (55)	0.476
	MB (%)	24 (13)	8 (14)	0.350
SPT				
	L-ACh-induced spasms (%)	43 (24)	12 (29)	0.539
	TOC (%)	15 (8)	5 (12)	0.474
	Focal spasms (%)	51 (28)	14 (33)	0.533
	MVS (No, %)	98 (152, 64)	28 (38, 74)	0.283
	ST elevation during SPT (%)	29 (16)	5 (12)	0.487
	Unavoidable use of NTG (%)	50 (32)	11 (27)	0.551
Follow-up				
	Follow-up period (M)	89 (38, 113)	87 (55, 108)	0.843
	No. of anginal attack/M	0 (0, 1) (No. = 135)	0 (0, 1) (No. = 35)	0.864
	No. coronary vasodilators	1.4 ± 0.9 (No. = 135)	1.6 ± 1.1 (No. = 35)	0.266
	Cardiac death (%)	1 (0.6)	0 (0)	0.622
	Readmission for heart (%)	26 (15)	7 (15)	0.726

Numbers were expressed as the numbers (percentage), and values were expressed as the mean ± standard deviation or median (interquartile range). CAD, coronary artery disease; CAG, coronary angiography; FH-CAD, family history of coronary artery disease; FMD, flow-mediated dilation; L-ACh, low dose of acetylcholine; M, months; MB, myocardial bridging; MVS, multivessel spasm; NID, nitroglycerin-induced dilation; No., number; TOC, total occlusion due to spasms; SPT, spasm provocation test; VA, variant angina; VSA, vasospastic angina.

## Data Availability

Not applicable.
